# SDHB immunohistochemistry for prognosis of pheochromocytoma and paraganglioma: A retrospective and prospective analysis

**DOI:** 10.3389/fendo.2023.1121397

**Published:** 2023-03-16

**Authors:** Tingwei Su, Yifan Yang, Lei Jiang, Jing Xie, Xu Zhong, Luming Wu, Yiran Jiang, Cui Zhang, Weiwei Zhou, Lei Ye, Guang Ning, Weiqing Wang

**Affiliations:** ^1^ Shanghai Key Laboratory for Endocrine Tumors, Shanghai Clinical Centre for Endocrine and Metabolic Diseases, Shanghai Institute of Endocrine and Metabolic Diseases, Ruijin Hospital, Shanghai Jiaotong University School of Medicine, Shanghai, China; ^2^ Department of Pathology, Ruijin Hospital, Shanghai Jiaotong University School of Medicine, Shanghai, China; ^3^ Laboratory for Endocrine and Metabolic Diseases of Institute of Health Science, Shanghai Jiaotong University School of Medicine and Shanghai Institutes for Biological Sciences, Chinese Academy of Sciences, Shanghai, China

**Keywords:** SDHB immunohistochemistry, pheochromocytomas and paragangliomas, tumor progression, prognosis, retrospective, prospective

## Abstract

**Introduction:**

Pheochromocytomas and paragangliomas (PCC/PGL) are rare neuroendocrine tumors and can secrete catecholamine. Previous studies have found that SDHB immunohistochemistry (IHC) can predict SDHB germline gene mutation, and SDHB mutation is closely associated with tumor progression and metastasis. This study aimed to clarify the potential effect of SDHB IHC as a predictive marker for tumor progression in PCC/PGL patients.

**Methods:**

We included PCC/PGL patients diagnosed in Ruijin Hospital, Shanghai Jiao Tong University School of Medicine from 2002 to 2014 for retrospective analysis and discovered that SDHB (-) staining patients had poorer prognoses. Then we examined SDHB protein expression by IHC on all tumors in the prospective series, which was composed of patients from 2015 to 2020 in our center.

**Results:**

In the retrospective series, the median follow-up was 167 months, and during follow-up, 14.4% (38/264) patients developed metastasis or recurrence, and 8.0% (22/274) patients died. Retrospective analysis revealed that 66.7% (6/9) of participants in the SDHB (-) group and 15.7% (40/255) of those in the SDHB (+) group developed progressive tumors (OR: 10.75, 95% CI: 2.72-52.60, P=0.001), and SDHB (-) was independently associated with poor outcomes after adjusting by other clinicopathological parameters (OR: 11.68, 95% CI: 2.58-64.45, P=0.002). SDHB (-) patients had shorter disease-free survival (DFS) and overall survival (OS) (P<0.001) and SDHB (-) was significantly associated with shorter median DFS (HR: 6.89, 95% CI: 2.41-19.70, P<0.001) in multivariate cox proportional hazard analysis. In the prospective series, the median follow-up was 28 months, 4.7% (10/213) patients developed metastasis or recurrence, and 0.5% (1/217) patient died. For the prospective analysis, 18.8% (3/16) of participants in the SDHB (-) group had progressive tumors compared with 3.6% (7/197) in the SDHB (+) group (RR: 5.28, 95% CI: 1.51-18.47, P=0.009), statistical significance remained (RR: 3.35, 95% CI: 1.20-9.38, P=0.021) after adjusting for other clinicopathological factors.

**Conclusions:**

Our findings demonstrated patients with SDHB (-) tumors had a higher possibility of poor outcomes, and SDHB IHC can be regarded as an independent biomarker of prognosis in PCC/PGL.

## Introduction

Pheochromocytomas and paragangliomas (PCC/PGL) are rare tumors originating from chromaffin cells and arising from the adrenal medulla and extra-adrenal sites. PGL are subdivided into sympathetic and parasympathetic PGL, depending on their location and catecholamine production. Previously, PCC/PGL were classified as benign or malignant based on the presence of metastatic lesions at nonchromaffin sites (1). However, since the 4th edition of WHO Endocrine Tumor Classification, the term ‘malignant’ was not used and was replaced by ‘metastatic’ in PCC/PGL, and all PCC/PGL have been considered as tumors with metastatic potential due to the lack of a histological system endorsed for defining the biological aggressiveness (2). This approach was maintained in the current WHO classification (3).

Given the fact that 15~40% of PCC/PGL patients will have recurrence (4, 5), and 5%~35% of PCC/PGL patients can develop metastasis (6–10), whose five-year survival ranges from 50% to 70% (11, 12), all PCC/PGL patients should receive at least ten years of follow-up and annual reexaminations (13), and high-risk patients should be offered lifelong annual follow-up. Current evaluation of the tumor progression potential of PCC/PGL is based on multifactorial risk assessments, including the presence of SDHB mutation, larger tumor size, extra-adrenal location, dopamine hypersecretion, and several histopathologic scoring systems (14, 15). The first scoring system was Pheochromocytoma of the Adrenal Gland Scaled Score (PASS), proposed by Thompson in 2002 (16), and then several groups formulated different scoring systems, including GAPP, ASES, and COPPs (17–19).

Approximately 40% of PCC/PGL carry a germline mutation in one of at least 20 genes (20). Among these germline mutations, SDHB mutation often displays an increased risk of metastasis and recurrence and is the strongest indicator of metastasis in PCC/PGL (21), rather than other subunits of SDH (22). The SDHB gene encodes for SDHB protein assembled in the mitochondria to form succinate dehydrogenase (SDH), a key respiratory enzyme, that has an essential role in cell energy production. With absent or abnormal SDHB, the mitochondrial complex II fails to assemble and loses its enzymatic activity, causing the accumulation of succinate, inhibiting α-KG-dependent dioxygenases, and resulting in a pseudo hypoxic state, hypermethylation, and subsequent invasive behavior (23).

As previously mentioned, SDHB mutation in PCC/PGL patients is associated with an increased risk of tumor progression, and several studies have shown that SDHB gene mutations can be detected by the loss of SDHB staining by immunohistochemistry (IHC) (24, 25), we hypothesized that loss of SDHB expression in PCC/PGL tumors can function as a risk factor for progressive tumors in the current study.

Remarkably, SDHB IHC becomes negative most commonly in the setting where is bi-allelic inactivity in any of the four SDH subunit genes (SDHA, SDHB, SDHC, and SDHD) (26), and loss of function of both alleles almost always occurs in the presence of a germline mutation in PCC/PGL, but it can also occur in somatic mutation isolated to neoplastic cells (27). In addition, hypermethylation of the SDHC promoter, resulting in the silencing of the SDHC gene and subsequent inactivity of the SDH complex, can also cause completely negative cytoplasmic staining for SDHB (28, 29).

Recent studies have shown that SDHB protein loss can be used as an IHC biomarker to independently predict the prognosis of PCC/PGL patients (30–32), and a modified GAPP system (M-GAPP) and COPPs scoring system also incorporates loss of SDHB staining for analyzing PCC/PGL risk (18, 33). Despite these above studies have included SDHB IHC in the investigation of the prognosis of PCC/PGL, no reliable prospective study or long-term follow-up for PCC/PGL focusing on SDHB IHC is available at present. In the present study, we analyzed the value of SDHB IHC for distinguishing between high-risk or low-risk of progressive tumors in large retrospective and prospective series in our center.

## Materials and methods

### Study populations

We performed a retrospective and prospective study of PCC/PGL patients diagnosed in Ruijin Hospital, Shanghai Jiao Tong University School of Medicine from 2002 to 2020 and collected their tumors after surgery in our center.

**Figure 1:**
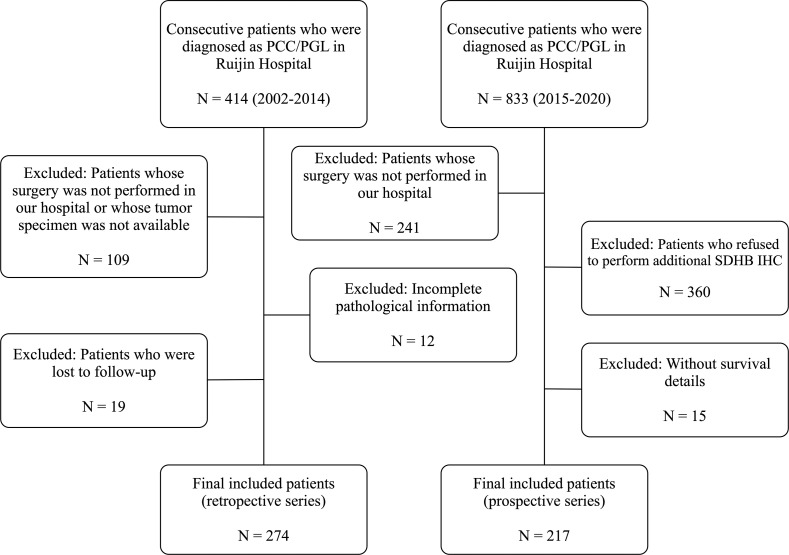
Flow diagram of the retrospective series and prospective series.

The retrospective study was composed of a series of 274 tumors from 274 patients who underwent an operation in our center from 2002 to 2014, including 195 PCC and 79 PGL, and this series was based on the population of our previous study (5). Patients in the retrospective study were selected according to the following inclusion criteria: histologically confirmed diagnosis of PCC/PGL and there was no perioperative mortality. At first, we included 414 patients in our retrospective study. The exclusion criteria were as follows: 1) Patients whose surgery was not performed in our hospital or whose tumor specimen was not available (n=109); 2) Incomplete pathological information (n=12); 4) Patients who were lost to follow-up (n=19). Thus 274 patients were finally included in the retrospective analysis (**Figure 1**).

The prospective study was performed on 217 tumors from 217 patients, which included 153 PCC and 64 PGL, and they received surgery in our center from 2015 to 2020. Patients in the prospective study were selected according to the same inclusion criteria as the retrospective study, and initially, 833 patients were included in the prospective analysis. The exclusion criteria were as follows: 1) Patients whose surgery was not performed in our hospital (n=241); 2) Patients who refused to perform additional SDHB IHC (n=360); 3) Without survival details (n=15) (**Figure 1**). Thus 217 patients were finally included in the prospective analysis.

Their baseline clinical characteristics, including age at diagnosis, sex, primary tumor location, primary tumor size, and catecholamine type were obtained by preoperational examinations. We have categorized the catecholamine-producing phenotype of PCC/PGL as adrenergic phenotype, in case the plasma metanephrine (MN), urinary adrenalin, or urinary dopamine levels exceeds the upper limits; noradrenergic phenotype, in case the normetanephrine (NMN) or urinary noradrenaline exceeds the upper limits without elevated MN/adrenaline/dopamine; or nonfunctioning phenotype, in case catecholamines were within the normal range, according to MNs, urinary adrenalin and dopamine levels. The postoperative pathological evaluation included TNM classification (categorized as I, II, III, or IV), and the TNM staging classification was based on the system set by the American Joint Committee on Cancer (AJCC) (34).

### IHC of SDHB

Formalin-fixed, paraffin-embedded tissue specimens were obtained and handled by standard surgical oncology procedures. Serial 5-μm sections were prepared and deparaffinized with xylene and gradient concentrations of alcohol (100%, 95%, 80%, and 75%) and then rehydrated. Immunoreactions of these slides were incubated overnight in a solution of SDHB antibody (rabbit polyclonal HPA002868; Sigma-Aldrich Corp, St Louis, MO, USA;1:500). Human adrenal gland tissue was used as the positive control and showed strong granular staining in the cytoplasm. And in the tumor, endothelial cells, sustentacular cells, and lymphocytes served as the internal positive control for each sample. For the retrospective series, SDHB IHC was carried out on all tumors in the Endocrine and Metabolic Diseases department of Ruijin Hospital. For the prospective study, SDHB IHC was consecutively performed in the Pathology department of Ruijin Hospital, allowing simultaneous detection of SDHB protein after surgery. All stained slides were analyzed blindly, by two independent observers (QW and JX), without any knowledge of the clinical data. The interpretation criteria used in this study followed the reported one (25). In order to determine whether SDHB IHC is negative, we rely on the consensus of the two observers. And only complete loss of SDHB staining was defined as SDHB immunonegative (–), otherwise, we judged it as immunopositive (+).

### Detection of SDHB germline mutation

In the retrospective population, all 274 patients underwent SDHB mutation analysis. DNA was collected from peripheral blood and isolated using standard procedure. SDHB mutation analysis was performed by commercial Sanger sequencing of PCR products.

### Follow-up procedure

Postoperative patients were followed up every six months for the first year after surgery, then annually. Laboratory examinations and abdominal computed tomography or magnetic resonance imaging were performed at every visit. All patients were followed up until 2022. The study was approved by the Ethics Committee of the Ruijin Hospital, Shanghai Jiao Tong University School of Medicine, and informed consent, which also permitted the collection of specimens and clinical data, was obtained from patients. Disease-free survival (DFS) time was defined as the date of surgery to the date of tumor progression, including recurrence or metastasis, confirmed by radiological and/or clinicopathological findings, and death. Overall survival (OS) time was defined as the interval between the date of surgery of and death or the latest follow-up.

### Statistical analysis

Descriptive statistics were presented as frequencies, percentages, median, and range. Comparisons of baseline characteristics between the two series were performed by t-test and Chi-square tests. The odds ratio (OR) was calculated by Logistic regression. Survival rates were compared by the Kaplan-Meier curves and differences in survival time were analyzed by log-rank tests. Hazard ratios were performed by univariate and multivariate Cox regression models. The risk ratio (RR) was calculated by Poisson regression with robust error variance. In multivariate analyses, we adjusted the baseline characteristics of PCC/PGL. All confidence intervals (CIs) were stated at the 95% confidence level. P<0.05 was considered statistically significant. The statistical data visualization was performed in the R 4.0.3 environment, using RStudio software, and all statistical analyses were performed by RStudio V 1.3.1093 (Boston, Massachusetts, USA) and SPSS Statistics version 28 (IBM, Armonk, NY, USA).

## Results

### Baseline characteristics of patients

In the retrospective series, the median follow-up duration was 167 months, and the median follow-up duration of the prospective series was 28 months. During follow-up, 38 of 264 (14.4%) patients developed metastasis or recurrence, and 22 patients died in the retrospective series, while in the prospective series, 10 of 213 (4.7%) patients developed metastasis or recurrences, and 1 patient died. Overall, 23 patients died during our follow-up. Compared with the retrospective series, the prospective population showed similar baseline characteristics, including gender, primary tumor location, and catecholamine type, but differed in the age of diagnosis, primary tumor size, and TNM classification (**Table 1**).

**Table 1:** Demographics and baseline characteristics of the study population.

	Retrospective n=274 (n%)	Prospective n=217 (n%)	P
Age at diagnosis (years)			
Median (range)	44 (11 to 79)	48 (16 to 77)	<0.001
Sex			
Female	156 (56.9)	115 (53.0)	
Male	118 (43.1)	102 (47.0)	0.435
Primary tumor location			
Adrenal	195 (71.2)	153 (70.5)	
Extra-adrenal abdomen/pelvis	75 (27.4)	62 (28.6)	
Extra-adrenal thorax	2 (0.7)	1 (0.5)	
Extra-adrenal head/neck	2 (0.7)	1 (0.5)	0.973
Primary tumor size (cm)			
<5	114 (41.6)	133 (61.3)	
≥5	160 (58.4)	84 (38.7)	<0.001
TNM classification			
Stage 1	82 (29.9)	93 (42.9)	
Stage 2	172 (62.8)	98 (45.2)	
Stage 3	10 (3.6)	22 (10.1)	
Stage 4	10 (3.6)	4 (1.8)	<0.001
Catecholamine type*			
Adrenergic subtype	149 (60.8)	125 (57.6)	
Noradrenergic subtype	79 (32.2)	83 (38.2)	
Nonfunctioning subtype	17 (6.9)	9 (4.1)	0.226
SDHB IHC			
Negative	12 (4.4)	17 (7.8)	
Positive	262 (95.6)	200 (92.2)	0.156
Newly discovered metastasis or recurrence			
Yes	38 (14.4)	10 (4.7)	
No	226 (85.6)	203 (95.3)	<0.001
Survival status			
Dead	22 (8.0)	1 (0.5)	
Alive	252 (92.0)	216 (99.5)	<0.001

*Based on 245 patients had catecholamine examination before surgery for retrospective analysis.

In the retrospective series, the median age at diagnosis of PCC/PGL was 44 years old, ranging from 11 to 79, and 56.9% (156/274) of individuals were female. There were 58.4% of patients had a larger (≥ 5cm) primary tumor size and 28.8% of primary tumors were located in extra-adrenal sites (75 in the abdomen/pelvis, two in the thorax, and two located in the head/neck region). Preoperative catecholamine levels were available in 89.4% (245/274) patients, including 60.8% (149/245) adrenergic subtype, 32.2% (79/245) noradrenergic subtype, and 6.9% (17/245) nonfunctioning subtype. TNM classifications were obtained in all patients, composed of 3.6% stage 4 tumors, 3.6% stage 3 tumors, 62.8% stage 2 tumors, and 29.9% stage 1 tumors. The SDHB staining was negative in 12 (4.4%) tumors.

In the prospective series, the median age at diagnosis was 48 years old, with a range of 16 to 77 years old, and 53.0% (115/217) of the patients were female. 38.7% of patients had primary tumors that were bigger (≥ 5 cm) in size, and 29.5% of primary tumors were of extra-adrenal origin, including 62 located in the abdomen/pelvis, one in the thorax, and one in the head/neck region. Preoperative catecholamine levels were available for all patients, and 57.6% were adrenergic subtype, 38.2% were noradrenergic subtype, and 4.1% were nonfunctioning subtypes. The percentages of stage 4 tumors, stage 3 tumors, stage 2 tumors, and stage 1 tumors by TNM classification, were 1.8%, 10.1%, 45.2%, and 42.9%, respectively. The SDHB staining was negative in 17 (7.8%) tumors.

### Retrospective study

Among the 274 patients, ten patients had metastatic lesions when they received an operation in our hospital, thus only 264 individuals were included in further analysis, except for the OS analysis. During follow-up, there were 46 patients that developed progressive tumors or died, six with SDHB (-) staining and 40 with SDHB (+) staining. 66.7% (6/9) of participants in the SDHB (-) group had progressive tumors or died compared with 15.7% (40/255) in the SDHB (+) group (OR: 10.75, 95% CI: 2.72-52.60, P=0.001). After adjusted by other baseline parameters, including primary tumor size, primary tumor location, age at diagnosis, sex, catecholamine type, and TNM classification, SDHB (-) was still associated with poor outcomes (OR: 11.68, 95% CI: 2.58-64.45, P=0.002).

**Figure 2:**
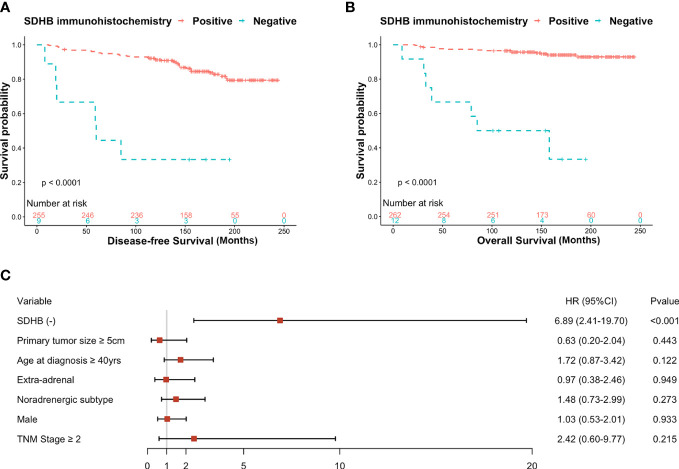
**(A)** Kaplan-Meier curves for DFS probability according to SDHB IHC in retrospective series, **(B)** Kaplan-Meier curves for OS probability according to SDHB IHC in retrospective series, **(C)** Forest plot of hazard ratios (HRs) by multivariate cox regression of DFS based on clinicopathological factors.

The Kaplan-Meier curve showed SDHB (-) patients were more likely to develop progressive tumors or died (P<0.001) (**Figure 2A**). The multivariate Cox regression analysis showed that SDHB IHC, even adjusted by other clinicopathological factors, was still a statistically significant independent predictor for DFS (HR: 6.89, 95% CI: 2.41-19.70, P<0.001) (**Figure 2C**). The median DFS in the retrospective series was 163 months (95% CI: 3-14 months), and the tumor-free rate of the 264 patients was 94.7% at five years and 90.9% at ten years. For the OS analysis, the Kaplan-Meier curve revealed that SDHB (-) patients had a higher possibility to die (P<0.001) (**Figure 2B**).

In addition, we compared the performance of SDHB IHC with SDHB germline mutation in predicting progressive tumors in our retrospective series, and the result is shown in **Table 2**. There were eight patients with SDHB germline mutation, and six of them were included in this comparison because two patients already had metastatic sites when they underwent surgery. Based on our result, we found that among the predictive index scores, the specificity of SDHB germline mutation was higher (99.08%) compared with SDHB IHC (98.62%). However, the sensitivity for predicting progressive behavior was higher in SDHB IHC (13.04%) compared with SDHB germline mutation (8.7%), and so was the negative predictive value.

**Table 2:** Predictive index scores of SDHB IHC and SDHB germline mutation in the retrospective study.

	Sen	Spe	NPV	PPV
SDHB germline mutation	8.70%	99.08%	83.72%	66.67%
SDHB IHC	13.04%	98.62%	84.31%	66.67%

Sen, sensitivity; Spe, specificity; NPV, negative predictive value; PPV, positive predictive value.

### Prospective study

**Figure 3:**
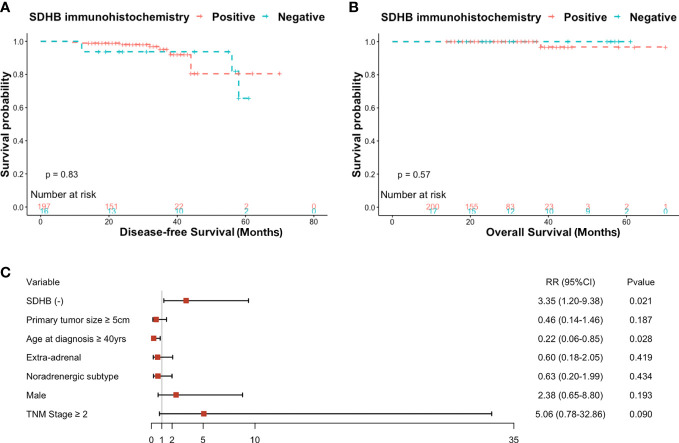
**(A)** Kaplan-Meier curves for DFS probability according to SDHB IHC in prospective series, **(B)** Kaplan-Meier curves for OS probability according to SDHB IHC in prospective series, **(C)** Forest plot of risk ratios (RRs) by multivariate generalized Poisson regression of DFS based on clinicopathological factors.

There were four patients who already had metastatic lesions when they were enrolled in the prospective study, thus only 213 individuals were included in the further analysis, except for the OS analysis. Ten patients had poor medical outcomes including recurrences, metastasis, and death during follow-up. 18.8% (3/16) of participants in the SDHB (-) group had progressive tumors or died compared with 3.6% (7/197) in the SDHB (+) group (RR: 5.28, 95% CI: 1.51-18.47, P=0.009). After regression analysis of mixed effects, including primary tumor size, primary tumor location, age at diagnosis, sex, catecholamine type, and TNM classification, SDHB IHC was still an independent predictor associated with prognosis (RR: 3.35, 95% CI: 1.20-9.38, P=0.021) (**Figure 3C**).

However, the Kaplan-Meier curve showed no difference in DFS or OS between the two groups (**Figures 3A, B**).

## Discussion

In this study, we included two series of PCC/PGL patients, composed of 274 tumors for retrospective study and 217 tumors for prospective study, to investigate the clinical significance of SDHB IHC. And this is a relatively large-scale study, using SDHB IHC of PCC/PGL tumors as a predictive factor to evaluate prognosis. We discovered that SDHB (-) patients had a much worse prognosis than SDHB (+) patients and that the lack of SDHB protein expression was an independent risk factor linked to progressive tumors. Both the retrospective analysis and the prospective investigation found that patients with SDHB (-) staining were more likely to experience adverse outcomes when considering the proportion of developing progressing tumors. Additionally, SDHB (-) patients had significantly shorter DFS and OS time than SDHB (+) individuals in survival analyses. However, we only found this tendency in the retrospective analysis, not the prospective study. Given the fact that between the retrospective series and prospective series, the median age at diagnosis, primary tumor size, and TNM classification were statistically different, which showed that in the retrospective series, patients were diagnosed with PCC/PGL at an earlier age, had a larger proportion of bigger tumors and late TNM stages. Thus, we thought that no statistical difference found in the prospective study was caused by the above clinicopathological differences and the mean follow-up duration of the prospective series was not as long as that of the retrospective series.

Previous studies which performed SDHB IHC also showed that the loss of SDHB protein is associated with poor prognosis (30, 32), and they discovered that patients with SDHB (-) tumors had considerably lower OS and DFS than those with SDHB (+) tumors. Compared to the research mentioned above, our study had a larger sample size and a longer follow-up time. However, there was a study that contradicted our conclusions, which found SDHB (+) was associated with shorter DFS in PCC patients (35), and we supposed it may be a result of different inclusion criteria for patients.

The SDHB protein is a catalytic subunit of succinate-ubiquinone oxidoreductase, also known as succinate dehydrogenase, or mitochondrial complex II, which links the electron transport chain and the Krebs cycle, and catalyzes succinate into fumarate. Other SDH-related genes (SDHA, SDHC, and SDHD) are autosomal inheritance and their corresponding proteins are assembled at the inner membrane of mitochondrial to form the SDH enzyme, except SDHAF2 encodes a protein that helps the insertion of FAD into SDHA (26). When SDH enzymatic activity decreased in SDHx-mutated tumors, SDHB expression levels by Western blotting and IHC were reduced as well (36). Comparing transcriptional and translational efficiency for indicating SDHB germline mutation, Chunzhang Yang and colleagues found that SDHB-related PCC/PGL tumors demonstrated unreduced mRNA expression but significantly declined protein expression than non-SDHB PCC/PGL (37), and most SDH-mutated tumors were negative for SDHB IHC, while non-SDH related tumors nearly all showed expression of SDHB by IHC (25). These results indicated that SDHB IHC is a favorable predictor for SDH-related tumors, better than transcriptional mRNA expression.

In addition to the loss of SDHB expression, we also found that larger tumor size, extra-adrenal location, noradrenergic phenotype, and earlier age at diagnosis were significantly associated with shorter DFS in PCC/PGL, in agreement with previous findings (14, 15, 38). Despite the fact that numerous studies have attempted to evaluate biomarkers for predicting PCC/PGL prognosis, there was no definite standard that can predict the clinical behavior of PCC/PGL, and the 5th series of the WHO Classification of Endocrine and Neuroendocrine Tumours does not support any of scoring systems but does not forbid their use in individual practices either (3). As our team analyzed before, SDHB mutation showed a higher odds ratio of metastasis (5), and previous studies had indicated that SDHB IHC can detect the presence of an SDH mutation (24, 25, 39). Based on our findings, the SDHB germline mutation demonstrated high specificity in the retrospective study compared with IHC; however, SDHB IHC was more sensitive and had a higher negative predictive value, which meant that more high-risk individuals and more low-risk individuals were divided into the poor prognosis group and the better prognosis group, respectively, and high-risk individuals would receive more stringent reexaminations and follow-up. And considering that many centers could not satisfy the need to undergo genetic tests for every PCC/PGL patient, this study can benefit PCC/PGL patients by using SDHB IHC to predict prognosis, as IHC is an easy procedure that could be performed in nearly all centers.

However, our study still has several limitations, such as the subjective definition of the IHC results which may decrease the predictive value of SDHB protein expression and this is an observational study, without any intervention, with a limited number of cases that had the main outcome. One of the reasons is that PCC/PGL are generally slow-growing tumors and metastasis commonly happens several years or even decades after the initial surgery, rendering investigation more difficult, thus a multicenter cohort study with a longer follow-up time is needed. The second reason may be the insufficient utilization of detection methods for metastasis or recurrence sites, such as 18F-FDOPA PET/CT and 68Ga-DOTATATE PET/CT. And at last, our proposed procedure can only be done after patients have undergone surgery and tumor tissue is available for pathological study.

In conclusion, we suggest that loss of SDHB protein can be regarded as a biomarker of adverse outcomes in PCC/PGL. Therefore, we recommend that patients with SDHB (-) tumors into consideration with more rigorous follow-up protocols, as they are more likely to develop progressive tumors.

## Data availability statement

The raw data supporting the conclusions of this article will be made available by the authors, without undue reservation.

## Ethics statement

The studies involving human participants were reviewed and approved by the ethics committee of the Ruijin Hospital, Shanghai Jiao Tong University School of Medicine. Written informed consent to participate in this study was provided by the participants’ legal guardian/next of kin. Written informed consent was obtained from the individual(s), and minor(s)’ legal guardian/next of kin, for the publication of any potentially identifiable images or data included in this article.

## Author contributions

GN and WW conceptualized the study. LJ, JX, YJ, CZ, WZ, and LY designed the study. XZ and LW collected the data. YY and TS did the analysis and drafted the article. All authors contributed to critical revisions and final approval of the article.

## References

[1] DeLellis RA LR Heitz PU Eng C. WHO classification of tumours: Pathology and genetics of tumours of endocrine organs. 3rd ed. Lyon, France: IARC (2004).

[2] Loyd R Osamura R Kloppel G Rosai J. WHO classification of tumours: pathology and genetics of tumours of endocrine organs. 4th ed. Lyon, France: IARC (2017).

[3] WHO Classification of Tumours Editorial Board. WHO classification of endocrine and neuroendocrine tumours. 5th ed. Lyon, France: IARC (2022).

[4] Bausch B Wellner U Bausch D Schiavi F Barontini M Sanso G. Long-term prognosis of patients with pediatric pheochromocytoma. Endocr Relat Cancer (2014) 21(1):17–25. doi: 10.1530/ERC-13-0415 24169644

[5] Zhong X Ye L Su T Xie J Zhou W Jiang Y. Establishment and evaluation of a novel biomarker-based nomogram for malignant phaeochromocytomas and paragangliomas. Clin Endocrinol (Oxf) (2017) 87(2):127–35. doi: 10.1111/cen.13357 28429830

[6] Dhir M Li W Hogg ME Bartlett DL Carty SE McCoy KL. Clinical predictors of malignancy in patients with pheochromocytoma and paraganglioma. Ann Surg Oncol (2017) 24(12):3624–30. doi: 10.1245/s10434-017-6074-1 28884434

[7] Ayala-Ramirez M Feng L Johnson MM Ejaz S Habra MA Rich T. Clinical risk factors for malignancy and overall survival in patients with pheochromocytomas and sympathetic paragangliomas: Primary tumor size and primary tumor location as prognostic indicators. J Clin Endocrinol Metab (2011) 96(3):717–25. doi: 10.1210/jc.2010-1946 21190975

[8] Neumann HPH Tsoy U Bancos I Amodru V Walz MK Tirosh A. Comparison of pheochromocytoma-specific morbidity and mortality among adults with bilateral pheochromocytomas undergoing total adrenalectomy vs cortical-sparing adrenalectomy. JAMA Netw Open (2019) 2(8):e198898. doi: 10.1001/jamanetworkopen.2019.8898 31397861 PMC6692838

[9] Buffet A Ben Aim L Leboulleux S Drui D Vezzosi D Libé R. Positive impact of genetic test on the management and outcome of patients with paraganglioma and/or pheochromocytoma. J Clin Endocrinol Metab (2019) 104(4):1109–18. doi: 10.1210/jc.2018-02411 30698717

[10] Wachtel H Hutchens T Baraban E Schwartz LE Montone K Baloch Z. Predicting metastatic potential in pheochromocytoma and paraganglioma: A comparison of PASS and GAPP scoring systems. J Clin Endocrinol Metab (2020) 105(12):e4661–70. doi: 10.1210/clinem/dgaa608 PMC7553245 32877928

[11] Hamidi O Young WF Jr. Gruber L Smestad J Yan Q Ponce OJ. Outcomes of patients with metastatic phaeochromocytoma and paraganglioma: A systematic review and meta-analysis. Clin Endocrinol (Oxf) (2017) 87(5):440–50. doi: 10.1111/cen.13434 PMC5854189 28746746

[12] Fishbein L Ben-Maimon S Keefe S Cengel K Pryma DA Loaiza-Bonilla A. SDHB mutation carriers with malignant pheochromocytoma respond better to CVD. Endocr Relat Cancer (2017) 24(8):L51–l5. doi: 10.1530/ERC-17-0086 28566531

[13] Plouin PF Amar L Dekkers OM Fassnacht M Gimenez-Roqueplo AP Lenders JW. European Society of endocrinology clinical practice guideline for long-term follow-up of patients operated on for a phaeochromocytoma or a paraganglioma. Eur J Endocrinol (2016) 174(5):G1–g10. doi: 10.1530/EJE-16-0033 27048283

[14] Lenders JWM Kerstens MN Amar L Prejbisz A Robledo M Taieb D. Genetics, diagnosis, management and future directions of research of phaeochromocytoma and paraganglioma: A position statement and consensus of the working group on endocrine hypertension of the European society of hypertension. J Hypertens (2020) 38(8):1443–56. doi: 10.1097/HJH.0000000000002438 PMC7486815 32412940

[15] Hamidi O Young WF Jr. Iñiguez-Ariza NM Kittah NE Gruber L Bancos C. Malignant pheochromocytoma and paraganglioma: 272 patients over 55 years. J Clin Endocrinol Metab (2017) 102(9):3296–305. doi: 10.1210/jc.2017-00992 PMC5587061 28605453

[16] Thompson LD. Pheochromocytoma of the adrenal gland scaled score (PASS) to separate benign from malignant neoplasms: A clinicopathologic and immunophenotypic study of 100 cases. Am J Surg Pathol (2002) 26(5):551–66. doi: 10.1097/00000478-200205000-00002 11979086

[17] Cho YY Kwak MK Lee SE Ahn SH Kim H Suh S. A clinical prediction model to estimate the metastatic potential of pheochromocytoma/paraganglioma: ASES score. Surgery (2018) 164(3):511–7. doi: 10.1016/j.surg.2018.05.001 29929757

[18] Pierre C Agopiantz M Brunaud L Battaglia-Hsu SF Max A Pouget C. COPPS, a composite score integrating pathological features, PS100 and SDHB losses, predicts the risk of metastasis and progression-free survival in pheochromocytomas/paragangliomas. Virchows Arch (2019) 474(6):721–34. doi: 10.1007/s00428-019-02553-5 30868297

[19] Kimura N Takayanagi R Takizawa N Itagaki E Katabami T Kakoi N. Pathological grading for predicting metastasis in phaeochromocytoma and paraganglioma. Endocr Relat Cancer (2014) 21(3):405–14. doi: 10.1530/ERC-13-0494 24521857

[20] Buffet A Burnichon N Favier J Gimenez-Roqueplo AP. An overview of 20 years of genetic studies in pheochromocytoma and paraganglioma. Best Pract Res Clin Endocrinol Metab (2020) 34(2):101416. doi: 10.1016/j.beem.2020.101416 32295730

[21] Assadipour Y Sadowski SM Alimchandani M Quezado M Steinberg SM Nilubol N. SDHB mutation status and tumor size but not tumor grade are important predictors of clinical outcome in pheochromocytoma and abdominal paraganglioma. Surgery (2017) 161(1):230–9. doi: 10.1016/j.surg.2016.05.050 PMC5164946 27839933

[22] Lee H Jeong S Yu Y Kang J Sun H Rhee JK. Risk of metastatic pheochromocytoma and paraganglioma in SDHx mutation carriers: A systematic review and updated meta-analysis. J Med Genet (2020) 57(4):217–25. doi: 10.1136/jmedgenet-2019-106324 31649053

[23] Dona M Neijman K Timmers H. MITOCHONDRIA: Succinate dehydrogenase subunit b-associated phaeochromocytoma and paraganglioma. Int J Biochem Cell Biol (2021) 134:105949. doi: 10.1016/j.biocel.2021.105949 33609747

[24] Papathomas TG Oudijk L Persu A Gill AJ van Nederveen F Tischler AS. SDHB/SDHA immunohistochemistry in pheochromocytomas and paragangliomas: A multicenter interobserver variation analysis using virtual microscopy: A multinational study of the European network for the study of adrenal tumors (ENS@T). Mod Pathol (2015) 28(6):807–21. doi: 10.1038/modpathol.2015.41 25720320

[25] van Nederveen FH Gaal J Favier J Korpershoek E Oldenburg RA de Bruyn EM. An immunohistochemical procedure to detect patients with paraganglioma and phaeochromocytoma with germline SDHB, SDHC, or SDHD gene mutations: A retrospective and prospective analysis. Lancet Oncol (2009) 10(8):764–71. doi: 10.1016/S1470-2045(09)70164-0 PMC4718191 19576851

[26] Gill AJ. Succinate dehydrogenase (SDH)-deficient neoplasia. Histopathology (2018) 72(1):106–16. doi: 10.1111/his.13277 29239034

[27] Giubellino A Lara K Martucci V Huynh T Agarwal P Pacak K. Urinary bladder paragangliomas: How immunohistochemistry can assist to identify patients with SDHB germline and somatic mutations. Am J Surg Pathol (2015) 39(11):1488–92. doi: 10.1097/PAS.0000000000000534 PMC4606469 26457353

[28] Killian JK Miettinen M Walker RL Wang Y Zhu YJ Waterfall JJ. Recurrent epimutation of SDHC in gastrointestinal stromal tumors. Sci Transl Med (2014) 6(268):268ra177. doi: 10.1126/scitranslmed.3009961 PMC7670881 25540324

[29] Haller F Moskalev EA Faucz FR Barthelmeß S Wiemann S Bieg M. Aberrant DNA hypermethylation of SDHC: A novel mechanism of tumor development in Carney triad. Endocr Relat Cancer (2014) 21(4):567–77. doi: 10.1530/ERC-14-0254 PMC4722532 24859990

[30] Blank A Schmitt AM Korpershoek E van Nederveen F Rudolph T Weber N. SDHB loss predicts malignancy in pheochromocytomas/sympathethic paragangliomas, but not through hypoxia signalling. Endocr Relat Cancer (2010) 17(4):919–28. doi: 10.1677/ERC-09-0316 20702724

[31] Gul AE Keser SH Barisik NO Gurbuz YS Sensu S Erdogan N. Succinate dehydrogenase complex iron sulfur subunit b (SDHB) immunohistochemistry in pheochromocytoma, head and neck paraganglioma, thoraco-Abdomino-Pelvic paragangliomas: Is it a good idea to use in routine work? Asian Pac J Cancer Prev (2021) 22(6):1721–9. doi: 10.31557/APJCP.2021.22.6.1721 PMC8418866 34181326

[32] Wang Y Chen D Pang Y Xu X Guan X Liu L. Value of immunohistochemical expression of apelin, succinate dehydrogenase b, chromogranin b, human epidermal growth factor receptor-2, contactin 4, and succinyl-CoA synthetase subunit beta in differentiating metastatic from non-metastatic pheochromocytoma and paraganglioma. Front Endocrinol (Lausanne) (2022) 13:882906. doi: 10.3389/fendo.2022.882906 35574028 PMC9096168

[33] Koh JM Ahn SH Kim H Kim BJ Sung TY Kim YH. Validation of pathological grading systems for predicting metastatic potential in pheochromocytoma and paraganglioma. PloS One (2017) 12(11):e0187398. doi: 10.1371/journal.pone.0187398 29117221 PMC5678867

[34] Jimenez C Libutti S Landry C Lloyd R McKay R Rohren E. Adrenal-neuroendocrine tumors. AJCC Cancer staging manual (2017) 8:919–27. doi: 10.1007/978-3-319-40618-3_77

[35] Kim HM Koo JS. Expression of glutamine metabolism-related and amino acid transporter proteins in adrenal cortical neoplasms and pheochromocytomas. Dis Markers (2021) 2021:8850990. doi: 10.1155/2021/8850990 33505538 PMC7806379

[36] Santi R Rapizzi E Canu L Ercolino T Baroni G Fucci R. Potential pitfalls of SDH immunohistochemical detection in paragangliomas and phaeochromocytomas harbouring germline SDHx gene mutation. Anticancer Res (2017) 37(2):805–12. doi: 10.21873/anticanres.11381 28179334

[37] Yang C Matro JC Huntoon KM Ye DY Huynh TT Fliedner SM. Missense mutations in the human SDHB gene increase protein degradation without altering intrinsic enzymatic function. FASEB J (2012) 26(11):4506–16. doi: 10.1096/fj.12-210146 PMC3475262 22835832

[38] Wang W Zhong X Ye L Qi Y Su T Wei Q. ERBB-2 overexpression as a risk factor for malignant phaeochromocytomas and paraganglinomas. Clin Endocrinol (Oxf) (2016) 84(6):822–9. doi: 10.1111/cen.13019 26749044

[39] Choi HR Koo JS Lee CR Lee JD Kang SW Jo YS. Efficacy of immunohistochemistry for SDHB in the screening of hereditary pheochromocytoma-paraganglioma. Biol (Basel) (2021) 10(7). doi: 10.3390/biology10070677 PMC8301322 34356532

